# Macrophages in osteoporotic fractures: from immunometabolic mechanisms to precision therapeutic approaches

**DOI:** 10.3389/fendo.2025.1698647

**Published:** 2025-11-21

**Authors:** Jiahui Xing, Haibo Li, Honggang Xia, Lilei Xia, Hongzhou Zhao

**Affiliations:** 1 Orthopedics and Traumatology Department of Integrated Traditional Chinese and Western Medicine, Tianjin Hospital, Tianjin, China; 2 Cardiothoracic Surgery Department Tianjin Hospital, Tianjin, China

**Keywords:** osteoporotic fractures, macrophages, immunometabolism, metabolic reprogramming, polarization, targeted therapy

## Abstract

Osteoporosis (OP) is a systemic bone disease characterized by reduced bone mass and deterioration of bone microarchitecture. Its critical complication, osteoporotic fractures (OPF), imposes a significant global disease burden. Macrophages, serving as central regulators within the osteoimmune microenvironment, dynamically modulate bone homeostasis and fracture healing through polarization (into pro-inflammatory M1 and reparative M2 phenotypes) and metabolic reprogramming. In OPF, OP-inducing factors (such as estrogen deficiency and aging) induce metabolic dysregulation in macrophages by disrupting the balance between glycolysis and oxidative phosphorylation (OXPHOS), causing aberrant succinate accumulation, and depleting NAD^+^ levels. This dysregulation disrupts the orderly transition from pro-inflammatory M1 to reparative M2 polarization, ultimately leading to insufficient inflammatory initiation in the early fracture phase and impaired osteogenic differentiation during later stages. Targeting this mechanism, innovative therapeutic strategies centered on macrophage metabolic reprogramming and polarization modulation are rapidly developing. These include nanocarriers for mitochondrial function restoration, bioactive coatings enabling time-programmed osseointegration, immunomodulatory smart hydrogels, and functionalized composite biomaterials. These strategies effectively promote osteoporotic bone regeneration by synergistically optimizing osteoimmune homeostasis and the osteoblast-osteoclast balance. This review systematically summarizes the immunometabolic mechanisms of macrophages in OPF and explores targeted intervention strategies, providing novel perspectives for the precision treatment of OPF.

## 1 Introduction

Osteoporosis (OP) is a systemic skeletal disorder marked by low bone mass, deteriorated bone microarchitecture, and consequently, increased bone fragility and fracture susceptibility (1). Its global prevalence is estimated at 19.7% (95% CI: 18.0%–21.4%) (2), rising to 21.7% among the elderly (95% CI: 18.8%–25.0%) (3). In 2019, OP incidence reached 41.5 million cases, reflecting a steady upward trend (4). Osteoporotic fractures (OPF) are a major complication of OP (5). Approximately 50% of women will experience at least one OPF during their lifetime (6). The associated annual global treatment costs are substantial, potentially reaching $25 billion USD (7).

Macrophages are highly heterogeneous immune cells that are key regulators of bone homeostasis within the osteoimmune microenvironment (8, 9). In response to local cues, they polarize into functionally distinct phenotypes (M1/M2) and release signaling molecules, including cytokines and exosomes. These signals modulate the activities of osteoblasts (OBs), osteoclasts (OCs), and bone marrow stromal cells (BMSCs) to maintain skeletal equilibrium (10–13). Furthermore, macrophages are indispensable for orchestrating bone repair following injury (14–16).

OPF is frequently complicated by delayed healing or non-union (17). While traditional theories of fracture repair have focused on biomechanics and OBs/OCs balance, emerging evidence underscores the central role of the osteoimmune microenvironment, where macrophages act as key orchestrators (18). Under the pathological state of osteoporosis, the impaired function of macrophages is an important mechanism of delayed healing of OPF (19). Therefore, deciphering the immunometabolic networks controlling macrophages in OPF and developing macrophage-targeted therapies to restore osteoimmune homeostasis may provide a promising avenue for addressing current treatment limitations and enabling precision intervention.

This review aims to systematically summarize the role of macrophages in the pathogenesis and progression of OPF. We will focus on the mechanisms underlying their immune polarization, metabolic reprogramming, and interactions with bone cells (OBs, OCs, BMSCs). We will elaborate on how OP-related pathological factors impair fracture healing by disrupting macrophage function. Furthermore, we will evaluate the potential and challenges of innovative therapies targeting macrophage immunometabolism for enhancing OPF repair. Ultimately, this review seeks to provide perspectives and a theoretical foundation for the future precision treatment of OPF.

## 2 Role of macrophages in bone homeostasis

Bone homeostasis depends on the dynamic balance between bone formation by OBs and bone resorption by OCs (20). Macrophages, as important immune effector cells, help maintain this equilibrium by directly influencing the activities of OBs, OCs, and BMSCs (21). Their functional impact on bone metabolism is largely determined by their polarization state, which can shift toward either the pro-inflammatory M1 or the anti-inflammatory M2 phenotype.

### 2.1 Regulation of OBs by macrophages

OBs are essential for bone formation (22). Macrophages influence OB differentiation and activity in a polarization-dependent manner through the secretion of various factors. M2 macrophages generally exert pro-osteogenic effects by releasing molecules such as BMP-2 and TGF-β1, which promote OBs differentiation and bone matrix mineralization via activation of the canonical Smad/Runx2 signaling pathway (23–26). In addition, M2-derived oncostatin M binds to the gp130 receptor and activates the JAK/STAT signaling pathway, thereby synergistically enhancing osteogenic differentiation (27). Specific M2 subsets (e.g., CD301b^+^ macrophages) also secrete IGF-1, promoting OBs differentiation via activation of the Akt/mTOR signaling pathway (28). Furthermore, M2 macrophages enhance OBs activity through chemokines (e.g., C-X-C motif chemokine ligand (CXCL) 3, CXCL6, CXCL14) and exosomes that modulate cytoskeletal and inflammatory pathways, and deliver osteogenic miRNAs such as miR-26a-5p and miR-21a-5p (29–33).

In contrast, M1 macrophages primarily inhibit OBs activity through the release of pro-inflammatory cytokines. Key effector molecules such as TNF-α and IL-1β suppress the expression of essential osteogenic transcription factors and inhibit the WNT/β-catenin signaling pathway, thereby impairing OBs differentiation and function (34–36). Although its role is complex, IL-6 in M1-dominant environments often indirectly suppresses osteogenesis, for example by upregulating TNF-α in OBs (37–40). Exosomes derived from M1 macrophages have also been implicated in the regulation of OB activity, though their precise mechanisms and functional distinctions from M2-derived exosomes remain to be fully elucidated (33, 41). It is also noteworthy that TNF-α exhibits a dual role: brief, low-level exposure can promote osteogenesis, while sustained, high-concentration exposure predominantly inhibits it (42).

### 2.2 Regulation of OCs by macrophages

OCs and macrophages share a common myeloid progenitor, which can lead to a competitive relationship during their differentiation. Macrophage polarization significantly influences osteoclastogenesis. While M1 macrophages are generally considered to promote osteoclastogenesis, their effects are highly context-dependent. Their key pro-inflammatory cytokine, TNF-α, strongly enhances RANKL-induced osteoclastogenesis and acts as an autocrine/paracrine factor in OCs formation (21, 43–46). IL-1β has also been shown to directly promote OCs formation and bone resorption in the presence of RANKL and M-CSF (47, 48). However, IFN-γ secreted by M1 macrophages inhibits osteoclastogenesis in *in vitro* RANKL-induced models (49), underscoring the context-dependent nature of M1-mediated regulation.

Conversely, M2 macrophages generally suppress osteoclastogenesis. They inhibit RANKL-induced OCs formation by interfering with TNF-α signaling and downregulating CSF2 expression (50). Characteristic anti-inflammatory cytokines, such as IL-10 and IL-4, directly impede osteoclast differentiation (10, 21). Furthermore, M2-derived exosomes serve as effective inhibitors by delivering miRNAs (e.g., miR-1227-5p) or modulating pathways such as STAT3 via CYLD (51, 52). Recent evidence indicates that these exosomes can metabolically reprogram osteoclast precursors—for example, by enhancing glutamine metabolism—and epigenetically downregulate osteoclastogenic genes, and may even promote their conversion into an M2-like phenotype. This establishes an important negative feedback loop that restrains bone resorption (53).

### 2.3 Crosstalk between macrophages and BMSCs

#### 2.3.1 Influence of macrophages on BMSCs

Macrophage polarization significantly influences the osteogenic differentiation of BMSCs. M2 macrophages promote osteogenic differentiation and mineralization of BMSCs through the secretion of factors such as BMP-2 and TGF-β1. These ligands activate the Smad/Runx2 pathway in BMSCs, leading to upregulated expression of key osteogenic markers including ALP, OCN, and COL1A1 (54–58). Additionally, specific miRNAs (e.g., miR-26a-5p, miR-486, miR-381) packaged within M2-derived exosomes enhance osteogenic gene expression and contribute to bone repair (32, 59, 60).

The influence of M1 macrophages on BMSCs is primarily dependent on the inflammatory state of the microenvironment (61). Under low inflammatory conditions, M1 macrophages have been observed to promote osteogenic differentiation and enhance bone mineralization in co-culture systems (62, 63). This promotive effect may be mediated through the induction of high autophagy levels in BMSCs, which facilitates their migration and osteogenic commitment (64, 65).

Conversely, under high-inflammatory conditions, classical M1 macrophages strongly inhibit osteogenesis. They secrete elevated levels of TNF-α, IL-1β, and IL-6, which induce sustained activation of the NF-κB pathway in BMSCs (66–68). Furthermore, M1 macrophages can transfer oxidatively damaged mitochondria to BMSCs, disrupting redox homeostasis in the stem cells and thereby impairing osteogenic differentiation (69). Additionally, exosomes derived from hypoxia-induced M1 macrophages (e.g., those containing miR-222) have been shown to significantly reduce BMSCs viability and migratory capacity while promoting apoptosis (70). This contrast illustrates the context-dependent nature of M1 macrophage influence on bone formation.

#### 2.3.2 Influence of BMSCs on macrophages

BMSCs are effective regulators of macrophage polarization. Under inflammatory conditions, BMSCs secrete factors such as prostaglandin E2 to promote macrophage transition from the pro-inflammatory M1 to the anti-inflammatory M2 phenotype, thereby enhancing the production of anti-inflammatory cytokines like IL-10 (71, 72). Furthermore, activated BMSCs can upregulate BMAL1 in macrophages via the KDM6B-BMAL1 axis, which suppresses the TLR2/NF-κB pathway, reduces pyroptosis, and ultimately lowers the M1/M2 ratio (73). BMSCs-derived exosomes—carrying molecules such as miR-27a-3p, miR-146a, and lncRNA-CAHM—promote M2 polarization while suppressing M1 polarization through inhibition of NF-κB signaling or direct targeting of downstream genes (74–77).

**Figure 1:**
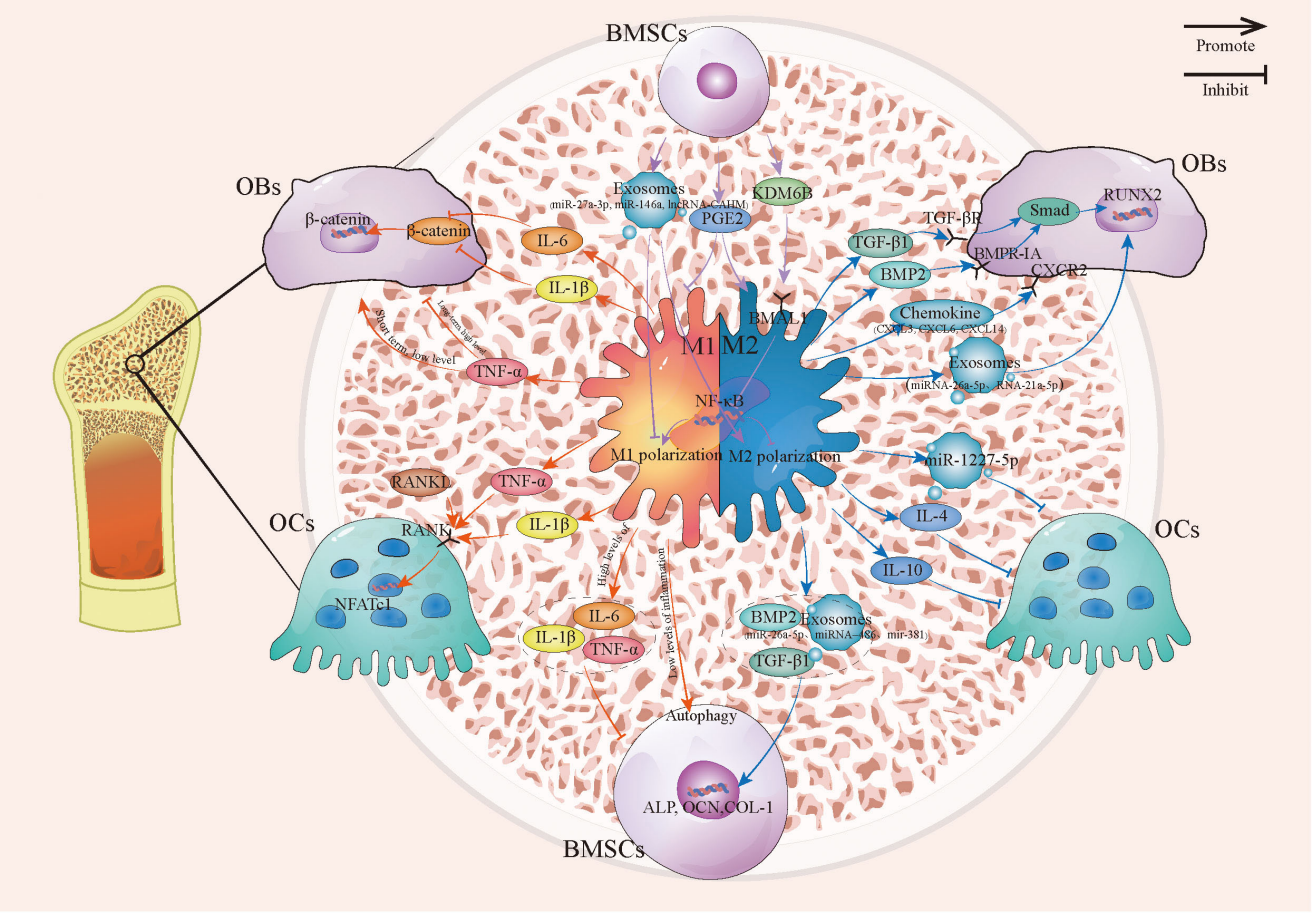
The connection between macrophages and OBs, OCs, and BMSCs. This image describes the relationship between macrophages and OBs, OCs, and BMSCs under different polarization states, including how M1 and M2 macrophages regulate OBs, OCs, and the mutual crosstalk between macrophages and BMSCs. BMSCs, Bone marrow stromal cells; OBs, Osteoblast; OCs, Osteoclast.

In summary, macrophages orchestrate bone remodeling through a complex network of cytokines, chemokines, and exosomes, dictated by their polarization state. Key pathways include BMP/Smad/Runx2 (osteogenesis), RANKL/RANK/OPG (osteoclastogenesis), and NF-κB (inflammation-mediated bone suppression). Typically, M2 macrophages promote bone formation by synergizing with OBs and BMSCs, while simultaneously inhibiting OCs activity. In contrast, M1 macrophages within inflammatory environments inhibit osteogenesis and potentiate osteoclastogenesis. This crosstalk is bidirectional; BMSCs provide critical feedback by secreting factors that promote macrophages toward the pro-regenerative M2 phenotype. Together, this dynamic feedback loop between macrophages, OBs, OCs, and BMSCs is fundamental to maintaining bone homeostasis (**Figure 1**), and its dysregulation is a pivotal immunometabolic mechanism driving OPF pathogenesis and progression.

## 3 Macrophages in OP

**Figure 2:**
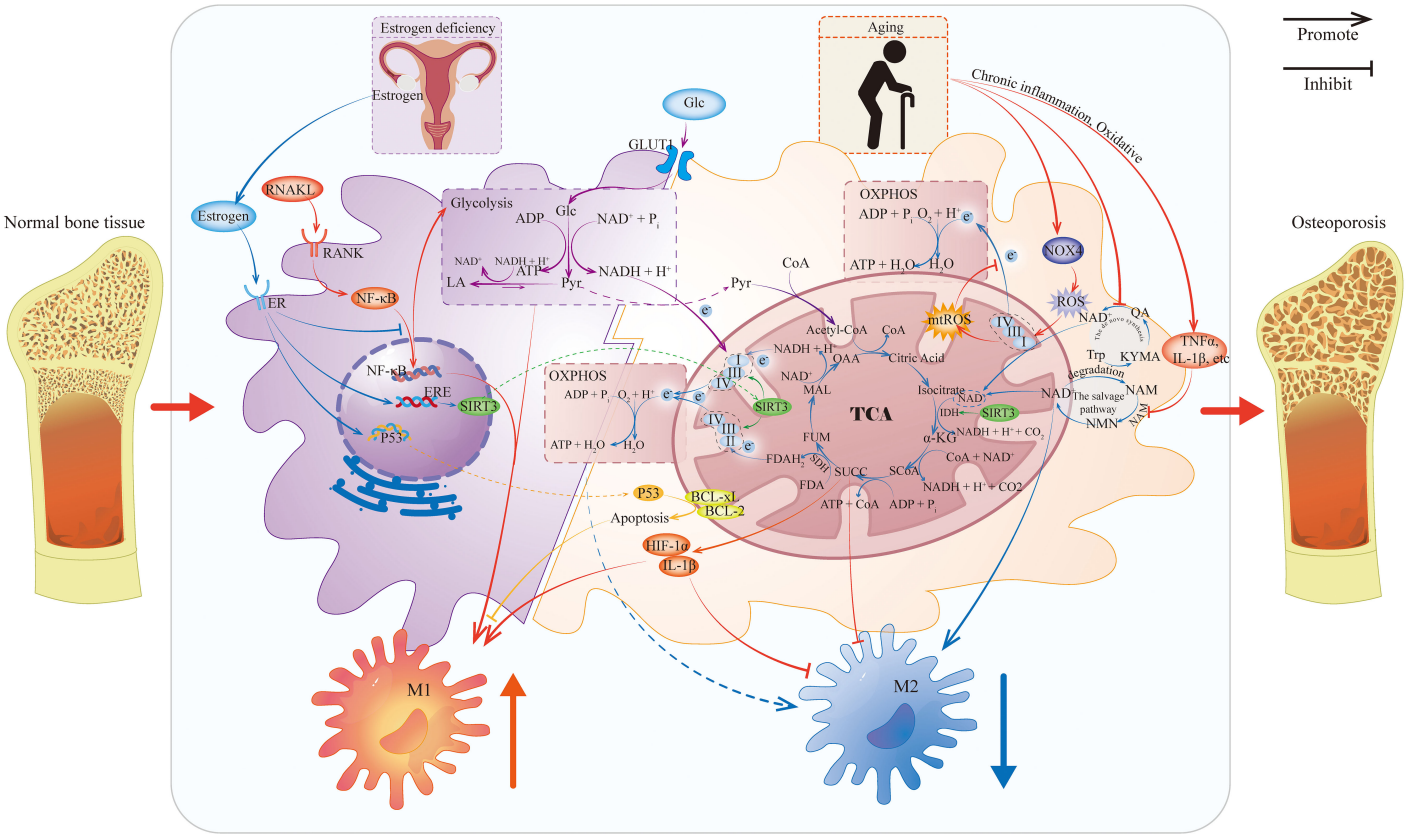
Metabolic reprogramming of macrophages caused by estrogen deficiency and aging. This figure illustrates how estrogen deficiency and aging affect macrophage metabolism and mediate macrophage polarization under OP. Because evidence of the role of gut microbiota and genetic mutations in the regulation of macrophages in OP is limited, it is not shown. IDH, Isocitrate Dehydrogenase; SDH, Succinate Dehydrogenase; CoA, Coenzyme A; OAA, Oxaloacetic Acid; MAL, Malic Acid; FUM, Fumaric Acid; SUCC, Succinic Acid; SCoA: Succinyl-CoA; α-KG, Alpha-Ketoglutaric Acid; TCA, Tricarboxylic Acid Cycle; Glc, Glucose; GLUT1, Glucose Transporter 1; Pyr, Pyruvic Acid; LA, Lactic Acid; ER, Estrogen Receptor; ERE, Estrogen Response Element; NAM, Nicotinamide; NMN, Nicotinamide Mononucleotide; NAMPT, Nicotinamide Phosphoribosyltransferase; Trp, Tryptophan; KYMA, Kynurenic Acid; QA, Quinolinic Acid.

OP is characterized by an imbalance in bone remodeling, resulting from enhanced bone resorption and reduced bone formation. Macrophages serve as central regulators of this process by modulating inflammatory status and bone homeostasis through immunometabolic reprogramming, which involves switching metabolic pathways and accumulating specific metabolites (78–81). Given the diverse etiologies of secondary osteoporosis, the following sections will adhere to the framework of primary OP to elucidate the role of the macrophage metabolism-polarization axis in OP pathogenesis (**Figure 2**).

### 3.1 Estrogen deficiency

The ovariectomized (OVX) mice model, which mimics postmenopausal osteoporosis, is widely used in related research. Studies using this model have shown an increased M1/M2 macrophage ratio in the bone marrow of osteoporotic mice. Under RANKL stimulation, aberrant differentiation of M2 macrophages into osteoclasts was observed, which contributed to enhanced bone resorption. Estrogen supplementation was found to reduce the M1 population and inhibit this macrophage-to-osteoclast transition (82, 83), indicating that estrogen has an important role in regulating macrophage polarization.

#### 3.1.1 Estrogen deficiency and RANKL-induced metabolic dysregulation

Mechanistically, estrogen deficiency impairs the ability of ERα to inhibit NF-κB p65 nuclear translocation, thereby enhancing cellular responsiveness to RANKL (83). Under physiological conditions, RANKL stimulation rapidly upregulates glucose transporters and enhances glycolytic enzyme activity in macrophages to meet their energy demands (84). Concurrently, RANKL induces high expression of aconitate decarboxylase 1 (ACOD1), which promotes the conversion of isocitrate to itaconate. Itaconate acts as an inhibitor of succinate dehydrogenase (SDH), leading to succinate accumulation and a disruption of the tricarboxylic acid (TCA) cycle by blocking the conversion of succinate to fumarate (85).

Succinate contributes to enhanced bone resorption through several mechanisms. First, it inhibits prolyl hydroxylases (PHDs), thereby preventing the degradation of hypoxia-inducible factor 1-alpha (HIF-1α). HIF-1α stabilization upregulates the secretion of pro-inflammatory cytokines such as IL-1β, fostering a microenvironment that supports M1 polarization (86, 87). Second, succinate activates macrophages via the SUCNR1 receptor, promoting their polarization toward the pro-inflammatory M1 phenotype and further facilitating their differentiation into OCs (88). Additionally, ACOD1-mediated itaconate accumulation and SDH inhibition impair electron transport chain (ETC) function by reducing electron flux from succinate oxidation, which worsens metabolic dysregulation (85).

#### 3.1.2 Estrogen deficiency and mitochondrial metabolic dysregulation

Upon binding to the ERα receptor on macrophages, estrogen upregulates the mitochondrial deacetylase SIRT3. SIRT3 enhances mitochondrial oxidative phosphorylation (OXPHOS) efficiency by deacetylating and activating mitochondrial ETC complexes (89, 90). At the same time, SIRT3 activates key TCA cycle enzymes, including isocitrate dehydrogenase and the pyruvate dehydrogenase complex. This activation facilitates the conversion of pyruvate and fatty acids to acetyl-CoA, promotes TCA cycle flux, and thereby enhances mitochondrial OXPHOS capacity (91, 92).

Furthermore, estrogen promotes the phosphorylation of p53 at Ser392 and facilitates its translocation to mitochondria. Within mitochondria, p53 binds directly to the anti-apoptotic proteins BCL-xL and BCL-2 through its DNA-binding domain. This interaction activates the mitochondrial apoptosis pathway, thereby reducing osteoclast formation (93, 94). In summary, by enhancing mitochondrial OXPHOS and suppressing glycolysis, estrogen attenuates M1 polarization of macrophages and limits osteoclastogenesis.

### 3.2 Aging

Aging is a major risk factor for chronic diseases, including metabolic disorders, cancer, and neurodegenerative diseases (95). In bone metabolism, aging is a primary driver of osteoporosis. As the global population ages, the associated disease burden is escalating (96, 97). Aging not only directly disrupts bone remodeling balance (98) but also accelerates bone loss through chronic inflammation and metabolic dysregulation (99, 100).

Aging remodels the immunometabolic program of macrophages, promoting a shift toward a pro-inflammatory phenotype. Experimental evidence shows a significant upregulation of M1 marker genes and an attenuated increase in M2 marker expression in bone marrow-derived macrophages from aged mice (101). Transcriptomic analysis of fracture callus further confirms that senescent macrophages exhibit dominant pro-inflammatory (M1) gene expression and dysregulation of immune-related networks (102). Critically, metabolic reprogramming is the central driver of this aging-associated polarization shift.

#### 3.2.1 Mitochondrial dysfunction drives pro-inflammatory polarization

Mitochondria are particularly vulnerable to aging-related changes. In macrophages, aging impairs ETC function and OXPHOS capacity, and is accompanied by a reduction in mitochondrial spare respiratory capacity (103, 104). The aging process involves upregulation of NADPH oxidase 4, resulting in reactive oxygen species (ROS) accumulation that further disrupts mitochondrial energy metabolism (105, 106). This metabolic disturbance triggers a compensatory shift toward glycolysis. However, aging is associated with an overall decline in glycolytic flux and reduced succinate levels, which exacerbates macrophage dysfunction (107). Impaired mitochondrial OXPHOS hinders effective reprogramming toward the anti-inflammatory (M2) phenotype (108). At the same time, accumulated ROS activates the NF-κB pathway, enhancing inflammatory responses and promoting polarization toward the pro-inflammatory (M1) phenotype (109, 110).

#### 3.2.2 NAD^+^ deficiency exacerbates energy metabolism imbalance

Aging is accompanied by a systemic decline in intracellular levels of the essential cofactor nicotinamide adenine dinucleotide (NAD^+^) (111–113). In macrophages under aging conditions, NAD^+^ synthesis becomes suppressed (113).

First, the *de novo* synthesis pathway is impaired. Macrophage *de novo* NAD^+^ synthesis originates from tryptophan metabolism via the kynurenine pathway (KP) (114). Upon immune challenge, indoleamine 2,3-dioxygenase 1 (IDO1) converts tryptophan into kynurenine, which is subsequently metabolized to quinolinic acid (115, 116). Quinolinic acid is then converted to nicotinic acid mononucleotide (NaMN) by quinolinic acid phosphoribosyltransferase, and NaMN is ultimately transformed into NAD^+^ via the Preiss-Handler pathway (117). Critically, in aging, innate immune challenges activate the upstream KP but restrict the downstream conversion of quinolinic acid to NAD^+^, resulting in dysfunction of the *de novo* synthesis pathway (114).

Second, the salvage pathway is impaired. This pathway serves as the primary source of NAD^+^ production during inflammatory stress. In it, NAD^+^ degradation products—primarily nicotinamide (NAM)—are converted to nicotinamide mononucleotide (NMN) by nicotinamide phosphoribosyltransferase (NAMPT). NMN is then transformed back into NAD^+^ by NMN adenylyltransferases (118–120). The expression of the rate-limiting enzyme NAMPT is induced by TNFα, IL-1β, LPS, IFNγ, and hypoxia—all factors that increase with age (121). Consequently, aging-associated inflammation can reduce NAD^+^ regeneration efficiency by dysregulating NAMPT.

NAD^+^ is an essential cofactor for the deacetylase activity of Sirtuin enzymes. The decline in NAD^+^ levels directly compromise SIRT activity (122, 123). Reduced SIRT3 activity impairs mitochondrial respiratory chain function and diminishes ATP synthesis, hindering the transition to the anti-inflammatory (M2) phenotype (124). Diminished SIRT3 activity also leads to impaired deacetylation of antioxidant enzymes like superoxide dismutase (SOD) 2, reducing cellular ROS-scavenging capacity and exacerbating oxidative stress (124, 125). Concurrently, decreased SIRT1 activity results in hyperactivation of the NF-κB pathway, promoting M1 polarization (126). In redox metabolism, NAD^+^ acts as a crucial electron carrier, accepting electrons from glycolysis and the TCA cycle to form NADH, which then transfers these electrons to the mitochondrial ETC for ATP production. Therefore, NAD^+^ deficiency exacerbates mitochondrial energy generation failure, ultimately disrupting macrophage phenotypic plasticity (127, 128).

#### 3.2.3 Autophagy defects amplify mitochondrial dysfunction and inflammation

Autophagy plays a critical role in regulating macrophage metabolism and polarization (129, 130). Aging is associated with a significant decline in macrophage autophagy, potentially linked to suppressed expression of the autophagy-related gene ATG5 (131, 132). Impaired mitophagy leads to the accumulation of dysfunctional mitochondria, exacerbating ROS production and OXPHOS deficiency (133, 134). Under specific conditions like hyperglycemia, mitochondrial-derived ROS can induce lysosomal dysfunction, further obstructing autophagic flux and promoting M1 polarization (135).

#### 3.2.4 Cellular senescence regulates polarization via metabolic reprogramming

Cellular senescence is frequently associated with downregulation of SIRT4 expression (136). In macrophages, SIRT4 modulates immune function by regulating branched-chain amino acid (BCAA) metabolism (137). BCAA catabolism depends on the branched-chain α-keto acid dehydrogenase (BCKDH) complex, whose activity requires functional dihydrolipoamide branched-chain transacylase E2 (DBT). SIRT4 ablation or downregulation leads to excessive itaconylation of DBT, reducing its enzymatic activity. This diminishes BCKDH activity, impairing BCAA breakdown and causing BCAA accumulation (137). The resulting BCAA buildup promotes pro-inflammatory polarization and impedes the transition to the anti-inflammatory phenotype (138, 139).

Although current studies have not directly established SIRT4 downregulation in senescent macrophages, the age-related decline in NAD^+^ may affect SIRT4 activity, potentially initiating this pathological cascade. Additionally, cellular senescence is marked by telomere damage; such telomere dysfunction disrupts mitochondrial metabolism in macrophages and activates the NLRP3 inflammasome through the PGC-1α/TNFAIP3 axis, thus promoting M1 polarization (140).

### 3.3 Gut microbiota

Gut dysbiosis represents a significant risk factor for OP (141, 142). Studies have revealed distinct compositional differences in gut microbiota between OP patients and healthy individuals (143), with proposed mechanisms involving microbial metabolites, immunoinflammatory modulation, and altered nutrient absorption (144–146). Although the precise mechanisms remain to be fully elucidated, the contribution of gut microbiota to OP pathogenesis has become increasingly evident, with macrophage polarization and metabolic reprogramming potentially serving as an intermediate link. Multiple microbial metabolites—including short-chain fatty acids (SCFA), bile acids, choline metabolites, indole derivatives, and vitamins—directly regulate macrophage polarization and metabolism (147).

SCFA including acetate, propionate, and butyrate, modulates bone metabolism through distinct mechanisms. For example, acetate reduces osteoclast numbers via T and B cells, while propionate and butyrate prevent OVX-induced bone loss by decreasing osteoclast formation (148). Although the regulatory effects of SCFA on macrophages have been documented in some studies—for instance, acetate enhances macrophage bactericidal activity (149). Propionate can promote the polarization of macrophages to anti-inflammatory M2 type by regulating the expression of transferrin receptor 1 and ferritin heavy chain 1 mediated by hypoxia-inducible factor (150). Butyrate can inhibit the M1 polarization of macrophages (151), the activation of inflammasomes, and reduce the production of osteoclasts, thereby improving osteolysis (152). However, the literature on the role of SCFA in regulating macrophage polarization and metabolic reprogramming in the context of OP is scarce and needs to be further explored.

In addition, bile acids derived from gut microbiota modulate macrophage polarization. For instance, under high-fat conditions, bile acids promote M1 polarization and pro-inflammatory cytokine production (153). They can also induce lipid peroxidation and suppress M2 polarization via the ROS/p38 MAPK/DGAT1 pathway, influencing disease processes such as acute myeloid leukemia (154). Trimethylamine oxide (TMAO), an oxidized metabolite of choline, enhances intracellular ROS levels and promotes osteoclast differentiation from macrophages (155). In contrast, indole-3-propionic acid inhibits osteoclast differentiation. Melatonin, a tryptophan-derived microbial metabolite, regulates TMAO metabolism and macrophage polarization, reduces inflammatory levels, and thereby ameliorates osteoporosis (156). These findings collectively indicate that gut microbiota metabolites can regulate both macrophage behavior and osteoporosis. However, direct evidence linking gut microbiota, their metabolites, macrophage polarization, and osteoporosis remains limited, suggesting that macrophages may act as an intermediate in the gut microbiota–OP axis, a hypothesis that requires further validation.

### 3.4 Genetic factors

Genetic predisposition is a major cause of primary OP. Mutations in genes such as *WNT1*, *PLS3*, and *XYLT2* directly impair osteocyte function, thereby contributing to OP pathogenesis (157). However, the mechanistic links between genetic determinants and OP development remain unexplored from the perspective of macrophage biology, representing a promising avenue for future investigation.

## 4 Macrophage polarization and metabolic reprogramming during fracture healing

**Figure 3:**
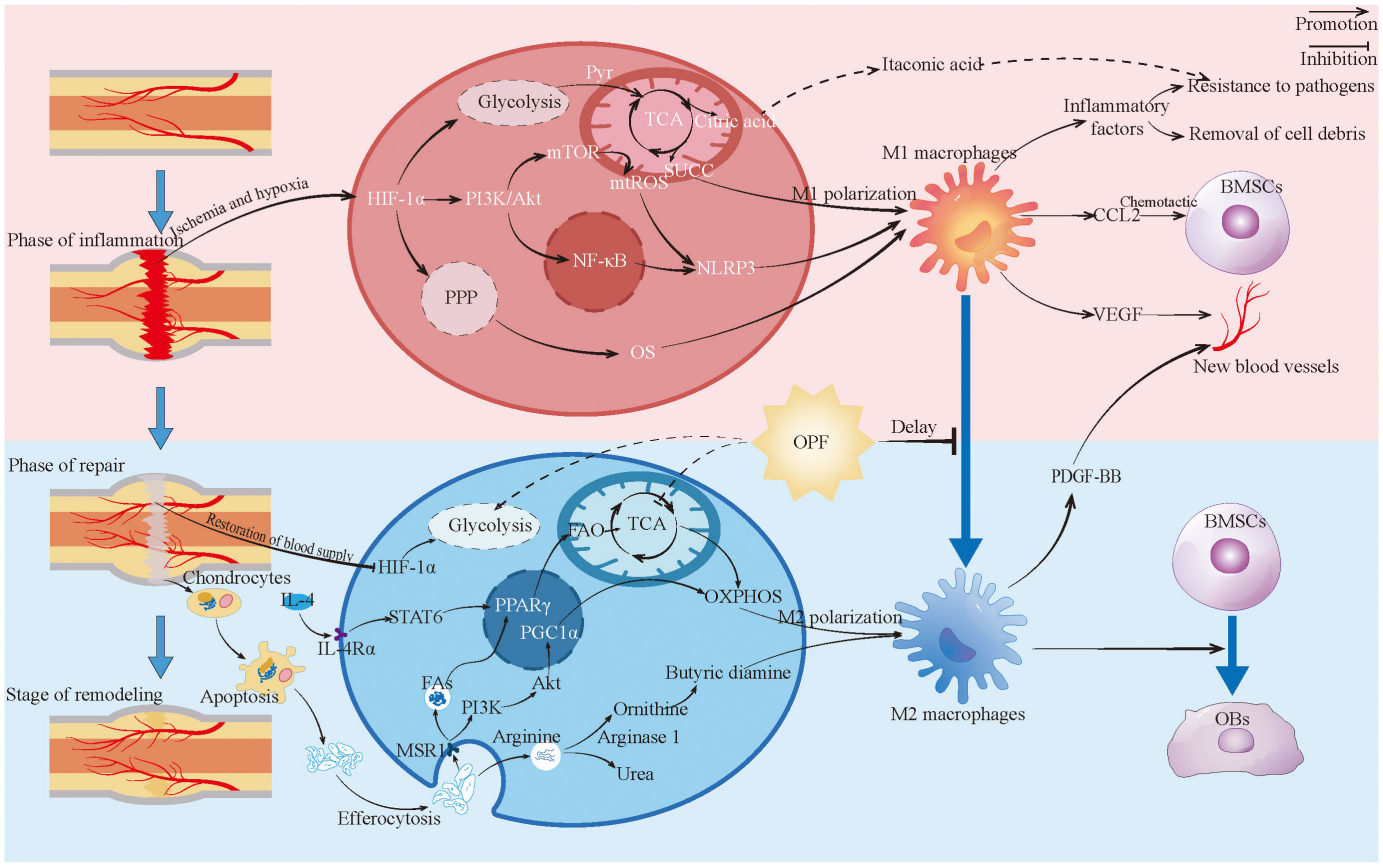
Macrophages in fracture healing. This figure depicts the changes and roles of macrophages during fracture healing, highlighting the metabolic reprogramming changes and contrasting with the characteristics of macrophages in the healing process of OPF. Pyr, Pyruvic acid; TCA, Tricarboxylic acid cycle; SUCC, Succinic acid; BMSCs, Bone marrow stromal cells; OPF, Osteoporotic fractures; PPP, Pentose phosphate pathway; MAR1, Macrophage scavenger receptor 1; Fas, Fatty acid; FAO, Fatty acid oxidation; OXPHOS, Oxidative phosphorylation; OBs, Bsteoblast.

Fracture healing is a complex process involving three overlapping phases: inflammatory, repair, and remodeling (158). Throughout these stages, macrophages play a crucial role by coordinating inflammatory responses, clearing debris, promoting angiogenesis, stimulating bone formation, and guiding tissue remodeling (159–161) (**Figure 3**).

### 4.1 Inflammatory phase

During the early inflammatory phase of fracture healing, disruption of the local blood supply creates a hypoxic microenvironment, leading to stabilization and elevated expression of HIF-1α (162). As a transcription factor, activated HIF-1α binds to promoters of multiple metabolic enzymes, upregulating glycolytic genes while suppressing OXPHOS (163, 164). This HIF-1α-driven glycolytic shift is a key mechanism promoting macrophage transition toward a pro-inflammatory phenotype (165). Although glycolysis generates pyruvate in macrophages, impaired TCA cycle flux leads to accumulation of citrate and succinate (166–168). Citrate is further metabolized to itaconate, which exerts antimicrobial effects, while succinate amplifies pro-inflammatory cytokine production in M1 macrophages (88, 169, 170). Under hypoxic conditions, HIF-1α overexpression also enhances the pentose phosphate pathway (PPP) in M1-like macrophages. The PPP generates nicotinamide adenine dinucleotide phosphate (NADPH), which helps modulate oxidative stress and sustain pro-inflammatory functions (165, 171–173). Furthermore, HIF-1α activates the NLRP3 inflammasome through the PI3K/AKT/mTOR signaling axis, thereby reinforcing the phenotypic effects of metabolic reprogramming (174).

M1 macrophages contribute to essential inflammatory responses by releasing cytokines such as TNF-α, IL-1β, IL-6, and CCL2, which aid in the clearance of pathogens and cellular debris (175). Through CCL2 secretion, they recruit BMSCs and promote prostaglandin E2 (PGE2) production via the COX-2–PGE2 axis. PGE2 then activates downstream signaling pathways in mesenchymal stem cells, facilitating osteogenic differentiation (62, 176). Concurrently, M1-derived vascular endothelial growth factor (VEGF) stimulates neovascularization, helping to alleviate hypoxia in the local microenvironment (177). Furthermore, within the early inflammatory milieu of fracture sites, IL-1β-stimulated M1 macrophages secrete inflammatory cytokines that enhance the expression and activity of nerve growth factor (NGF). By binding to tropomyosin receptor kinase A (TrkA) receptors, NGF promotes reinnervation, thereby supporting neural regeneration and functional recovery during bone repair (178–180).

### 4.2 Repair and remodeling phase

During the repair and remodeling phase, macrophages predominantly polarize toward the M2 phenotype (177). This phenotypic shift begins as early as the late inflammatory stage of fracture healing. The transition from M1 to M2 polarization appears to be facilitated by neovascularization that alleviates hypoxic conditions, leading to downregulation of glycolytic enzymes and restoration of OXPHOS in macrophages (177, 181, 182). Pro-inflammatory cytokines released by M1 macrophages during inflammation recruit immune cells; subsequently, microenvironmental cells (e.g., Th2 lymphocytes or BMSCs) secrete IL-4 and IL-10, modifying the immunoinflammatory milieu to further promote M2 polarization (183, 184). IL-4 activates the STAT6 pathway via IL-4Rα, inducing mitochondrial fatty acid oxidation (FAO) in M2 macrophages and upregulating the expression of CD36, CPT1, and PPARγ. This enhances mitochondrial respiratory chain activity and supports OXPHOS functionality (185, 186).

During endochondral ossification, macrophages phagocytose apoptotic chondrocytes, resulting in metabolic and polarization changes—a process referred to as efferocytosis (187–189). Studies have shown that apoptotic bodies derived from M2 macrophages contribute to regulating the M1/M2 balance (190). During efferocytosis, arginine from apoptotic cells is hydrolyzed by arginase 1 to produce ornithine and urea; ornithine is then catalyzed by ornithine decarboxylase (ODC) to generate putrescine, which enhances efferocytic efficiency and induces anti-inflammatory gene expression in macrophages (191, 192). Concurrently, the expression of macrophage scavenger receptor (MSR1) is upregulated. MSR1 activates the PI3K/AKT pathway, upregulates PGC1α expression, enhances mitochondrial OXPHOS, and promotes metabolic reprogramming along with M2 polarization (193). Moreover, fatty acids released from apoptotic chondrocytes can be taken up by macrophages via MSR1, subsequently activating PPAR-γ. This promotes fatty acid oxidation (FAO) in macrophages and stimulates BMP7 production, which facilitates osteogenic differentiation of BMSCs (194). These mechanisms act together to regulate macrophage metabolic reprogramming and polarization, thus supporting fracture healing.

In addition, M2 macrophages upregulate arginase-1 activity, shifting arginine metabolism away from the iNOS-dependent pathway—characteristic of M1 macrophages—and toward the synthesis of polyamines and proline. This metabolic shift promotes collagen deposition, cell proliferation, and angiogenesis (195, 196). Finally, PDGF-BB derived from M2 macrophages helps maintain neovascular stability, thereby supporting osteogenesis and nutrient delivery during bone remodeling (197).

Collectively, macrophages coordinate fracture healing through temporally regulated metabolic reprogramming and phenotypic switching (177). Initially, macrophages polarize toward the M1 phenotype via glycolytic metabolism, which enables clearance of necrotic debris, release of pro-inflammatory and chemotactic factors for BMSC recruitment, initiation of bone repair, and VEGF-mediated angiogenesis (198). As inflammation resolves, macrophage metabolism shifts toward OXPHOS, accompanied by a phenotypic transition from M1 to M2 (199). This transition further promotes osteogenic differentiation and mineral deposition by BMSCs, along with PDGF-BB release that stabilizes vasculature and consolidates newly formed bone (18). The metabolic plasticity of macrophages thus constitutes a regulatory hub for adapting to microenvironmental changes during bone regeneration (18).

### 4.3 Macrophages in OPF healing

In OPF healing, macrophage metabolism and polarization are disrupted by multiple pathological factors, leading to delayed union or nonunion (200). As described in earlier sections, estrogen deficiency impairs ERα-mediated inhibition of NF-κB p65 nuclear translocation, enhancing macrophage responsiveness to RANKL (83). RANKL stimulation induces high expression of ACOD1, promotes itaconate accumulation, and inhibits SDH, thereby disrupting the TCA cycle and leading to succinate accumulation (85). The accumulated succinate activates macrophages via the SUCNR1 receptor, driving polarization toward the pro-inflammatory M1 phenotype while suppressing OXPHOS, which is required for M2 polarization (88).

Experimental evidence supports this mechanism. Compared with the control group, the population of M2 macrophages in the callus of OVX mice was substantially reduced at 14 days post-fracture (DPF), concurrently showing decreased IL-4 secretion and a marked increase in IL-6 expression (201).

Aging exacerbates macrophage dysfunction through NAD^+^ deficiency, impaired autophagy, and cellular senescence, collectively leading to mitochondrial respiratory chain dysfunction, elevated oxidative stress, and enhanced inflammation—all of which favor M1 polarization. In aged rats with senile osteoporosis (SOP), transcriptomic analyses revealed significant upregulation of inflammation-related genes in macrophages. Serum levels of pro-inflammatory factors (IL-6 and TNF-α) were more than threefold higher than in young rats, whereas anti-inflammatory mediators such as IL-10 were markedly reduced (202, 203). These findings further support the concept that aging disrupts metabolic reprogramming and polarization in macrophages, thereby impairing osteoporotic fracture healing.

However, targeted studies examining the specific metabolic alterations in macrophages within the OPF microenvironment remain relatively limited. Key unresolved questions include whether macrophages in OPF exhibit metabolic dysregulation similar to that observed in osteoporosis alone, or whether these alterations are further exacerbated in the fracture context. Elucidating these mechanisms represents an important direction for future research on macrophage-centered interventions for OPF.

## 5 Therapeutic strategies targeting macrophages

As core regulators of the OPF immune microenvironment, macrophages directly orchestrate bone repair progression through their metabolic reprogramming and phenotypic polarization. Recent therapeutic advances in targeted macrophage therapies—leveraging innovative biomaterial design and delivery technologies—are demonstrating significant efficacy in modulating inflammatory responses and restoring osteoblast-osteoclast equilibrium.

**Figure 4:**
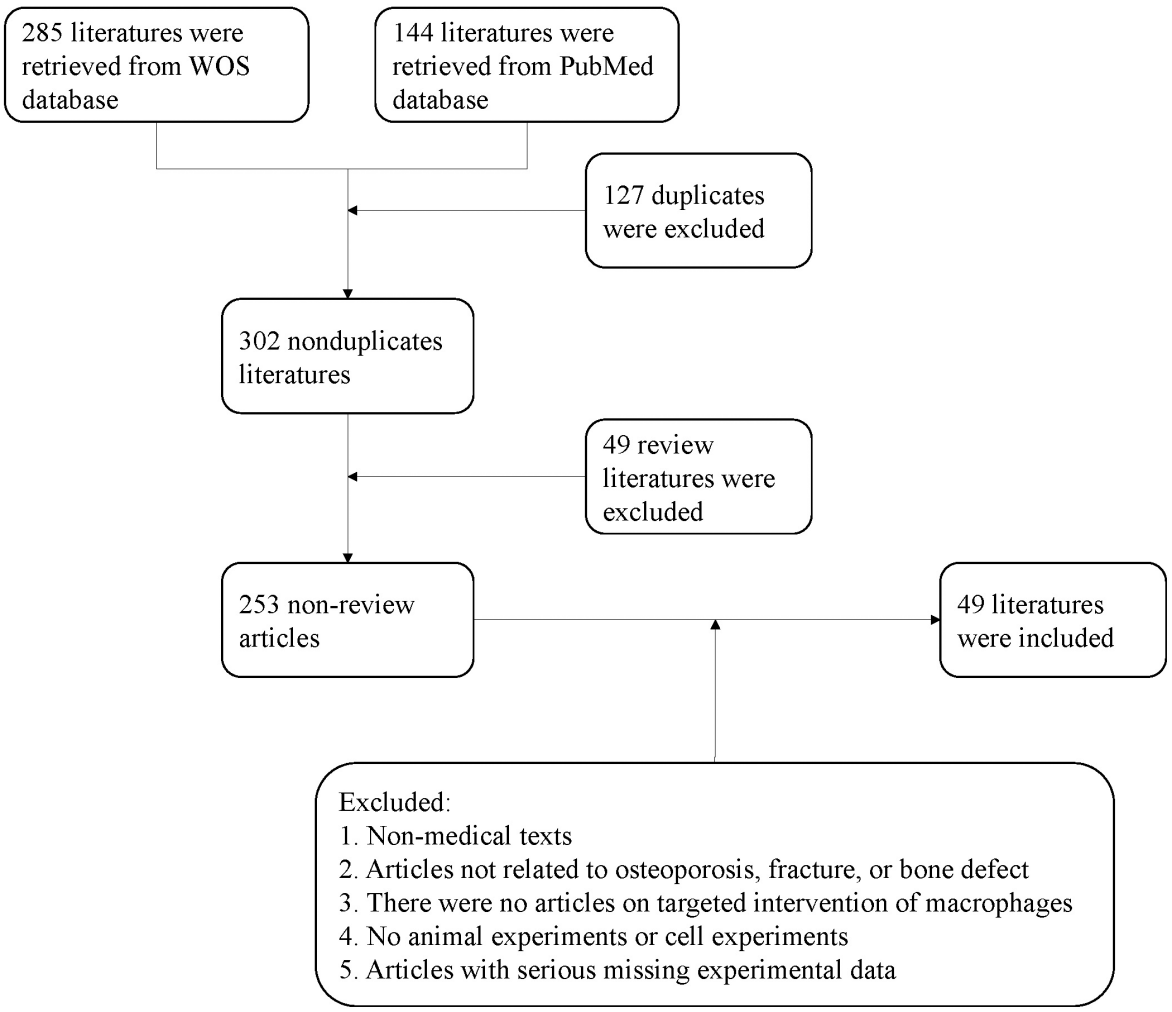
Flow chart of literature screening.

To identify relevant therapeutic strategies, we conducted a literature search of the PubMed and Web of Science (WOS) databases using the following keywords: “Macrophages,” “Osteoporosis,” “Fracture,” “Bone defect,” “Bone healing,” “Fracture healing,” “Bone repair,” “Bone regeneration,” and “Osteogenesis.” The search was limited to publications from the last decade, up to July 4, 2024. After excluding review articles and irrelevant studies, 49 publications were included in the analysis (**Figure 4**).

**Figure 5:**
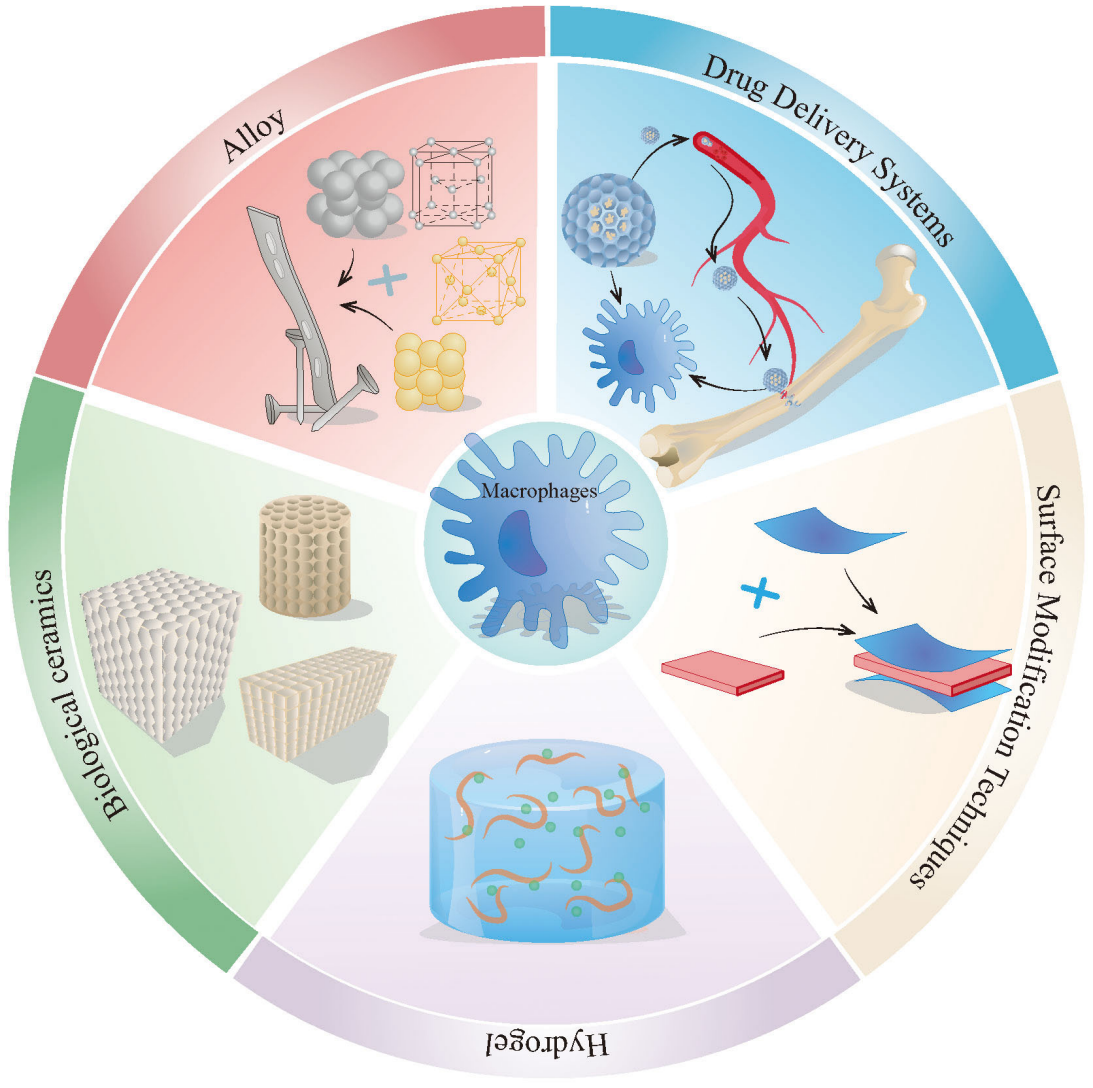
The method of treating osteoporotic fractures and bone defects by targeting macrophages. This figure describes the main approaches to target macrophages for the treatment of osteoporotic fractures and bone defects, including drug delivery systems, surface improvement technologies, hydrogels, alloys, bioceramics, etc. The alloy material in the picture uses the lattice of Zn and Cu. The actual alloy material may be made of other metals or more metals, and the lattice may not be the same as in the picture.

Based on the retrieved literature, this section systematically describes recent advances in drug delivery systems, surface modification technologies, hydrogels, and related biomaterials (**Figure 5**), with a focus on their mechanisms of action, therapeutic benefits, and translational challenges. The primary effects of different types of biomaterials on macrophage behavior are summarized in **Table 1**.

**Table 1:** Table of macrophage regulation strategies in osteoporotic bone repair based on biomaterials.

Material category	Core macrophage-targeting mechanisms	Overall therapeutic impact
Drug Delivery Systems	• Metabolic reprogramming (e.g., succinate/HIF-1α, mitochondrial function)	Precise spatiotemporal control over macrophage phenotype, promoting an anti-inflammatory and pro-regenerative microenvironment.
	• Suppression of pro-inflammatory signaling (e.g., NF-κB)	
	• Direct induction of M2 polarization	
Surface Modification	• Ion-mediated immunomodulation (e.g., Sr^2+^, Zn^2+^, Mn^2+^ via PI3K/Akt, NF-κB)	Sustained, surface-driven immunomodulation to enhance osseointegration and mitigate early-stage complications.
	• Metabolic regulation (e.g., citrate-induced glycolysis inhibition)	
	• Anti-inflammatory drug/coating release	
	• Temporal regulation of M1-to-M2 transition	
Hydrogels	• Potent ROS scavenging and antioxidant upregulation	Dynamic response to the injury microenvironment, effectively resolving oxidative stress and inflammation.
	• Anti-inflammatory polarization (e.g., via TLR4/NF-κB inhibition)	
	• Recruitment and instruction of host cells via released factors	
Bioceramics	• Immunomodulation via ionic release or physical cues (e.g., piezoelectricity)	Creation of a pro-osteogenic immune environment that supports bone formation and angiogenesis.
	• Inhibition of NF-κB signaling and RANKL-induced osteoclastogenesis	
Alloy Materials	• M1 suppression via ion release (e.g., Zn^2+^, Cu^2+^) and NF-κB inhibition	Integration of favorable mechanical properties with inherent immunomodulatory, antibacterial, and osteogenic effects.
	• Activation of pro-regenerative pathways (e.g., Wnt/β-catenin)	
Other Materials	• M2 polarization via ion release or bioactive compounds	Resolution of inflammation and oxidative stress through diverse pharmacological and material-based mechanisms.
	• Activation of cytoprotective pathways (e.g., Nrf2/HO-1/GPX4)	
	• Modulation of inflammatory signaling (e.g., TLR/MyD88)	

### 5.1 Drug delivery systems

Drug delivery systems use carriers to achieve active or passive targeting of macrophages, enabling precise modulation of their metabolism and polarization while improving drug bioavailability (**Table 2**).

**Table 2:** Drug delivery system.

Material	Carrier	Drug loading	Response	Cell model	Animal models		Macrophages	Osteoclast differentiation	Osteogenic differentiation	Inflammation and OS	Others	Ref.
ZIF-H_2_S-SDSSD NPs	ZIF-8 NPs, SDSSD	H_2_S	PH	BMM, BMSCs	OVX mice	Fracture of femur	M1↓, M2↑	Ctsk, Mmp9, Nfatc1, Atp6v0d2↓	RUNX2, SP7, OCN, OPN↑	IL-1β, IL-6↓; IL-10, TGF-β↑; mtROS↓	/	(204)
PLLA-MSN@VGX-1027	PLLA, MSN	VGX-1027	/	BMM, BMSCs	OVX mice	Femoral drilling defects	M2↑	/	RUNX2↑	TNF-α, IL-1α, IL-6↓; IL-10, TGF-β, Arg-1; O2-/·OH/H2O2↓	/	(205)
GMA MSs	GelMA	Mg, ALN	PH	RAW264.7, MC3T3-E1	OVX mice	Skull defect	M1↓, M2↑	Trap↓	ALP, OCN, OPN↑	TNF-α, IFN-γ↓ IL-10, TGF-β↑; ROS↓	/	(206)
MBNP-IP	MBNP	IP	/	RAW264.7	OVX rabbit	Defect of femur	M1↓	/	/	/	/	(207)
NanoMBG-Ips	MNPs	IP	/	EPCs, RAW 264.7	/	/	VEGFR2 is promoted by M2	/	/	/	VEGFR2 ↑	(208)
RSV@DTPF	DTPF	RSV	ROS	RAW264.7	OVX SD rats	Periodontal defect	M1↓, M2↑	Acp, Mmp9, Ctsk, Traf6↓	RUNX2, COL-1↑	TNF-α↓, IL-10↑, ROS↓	/	(209)
MZIF-8-PDA@Pta	MZIF-8-PDA	Melatonin, ZIF-8 NPs	/	RAW264.7, BMSCs	OVX SD rats	Defect of femoral condyle	M1↓, M2↑	/	RUNX2, OCN, BMP2, ALP↑	TNF-α, IL-6↓; IL-4, IL-10↑	/	(210)
IL-4/BEVs-BP@GelMA	GelMA	IL-4, BEVs-BP	/	RAW 264.7, VECs, BMSCs	OVX mice	Fracture of femur	M2↑	/	RUNX2, OCN, BMP2, COL-1↑	TNF-α, IL-1β↓; IL-4, TGF-β↑	VEGF, CD31↑	(211)
Gastrodin-functionalized scaffolds	PU/n-HA	Gastrodin	/	/	OVX SD rats	Defect of femur	M2↑	/	/	TNF-α, IL-1β↓	/	(213)
Fg@SCS NPs@rhBMP-2	SCS NPs	rhBMP-2	/	BMSCs	OVX mice	Fracture of femur	M1↓, M2↑	/	COL-1, OSX, TGF-B1, RUNX2, BMP2↑	/	/	(212)

OVX, Ovariectomized; NPs, Nanoparticles; BMMs, Bone marrow macrophages; BMSCs, Bone marrow stromal cells; MSN, Mesoporous silica Nanoparticles; PLLA, Poly-L-lactic acid; ALN, Alendronate; MBNP, Mesoporous bioactive Nanoparticles; IP, Ipriflavone; MNPs, Mesoporous nanospheres; DTPF, Distearoylphosphatidylethanolamineh-Tioktal-Polyethylene glyco-Folate; RSV, Resveratrol; ↓, Down-regulate or inhibit, ↑, Up-regulate or promote (The meanings of ↓ and ↑ in the subsequent table are the same as here).

#### 5.1.1 Regulating metabolic reprogramming

Metabolic reprogramming acts as an intrinsic driver of macrophage phenotypic polarization. Targeting this process to modulate macrophage function offers a promising strategy for improving fracture healing under osteoporotic conditions.

For example, Qin et al. developed bone-targeted nanoparticles (ZIF−H_2_S−SDSSD) functionalized with SDSSD peptide for osseous delivery. These particles remain stable under physiological pH (7.4) with minimal Zn^2+^ release, but degrade under acidic conditions to release H_2_S and Zn^2+^ ions, achieving responsive drug release. The released H_2_S and Zn^2+^ alleviate inflammation by reducing succinate accumulation in the TCA cycle and suppressing HIF-1α expression. H_2_S also protects mitochondrial function by decreasing mitochondrial reactive oxygen species (mtROS) production. *In vitro*, ZIF-H_2_S-SDSSD promotes macrophage repolarization from the M1 to the M2 phenotype, downregulating pro-inflammatory mediators while upregulating anti-inflammatory factors. *In vivo*, the treatment alters the macrophage composition at fracture sites—decreasing CD86^+^ M1 macrophages and increasing CD206^+^ M2 macrophages—and reprograms cellular metabolism by attenuating glycolysis, enhancing TCA cycle flux, and reducing succinate accumulation. Additionally, the nanoparticles suppress osteoclast-related genes (e.g., *Ctsk*, *Mmp9*) and activate osteogenic markers (e.g., *Runx2*, *OCN*), significantly improving osseointegration (204).

Wang et al. developed poly (L-lactic acid) mesoporous silica nanoparticle (PLLA-MSN@VGX-1027) microspheres targeting mitochondrial function for immunomodulation. *In vitro*, the released VGX-1027 upregulates key proteins regulating mitochondrial dynamics (MFN2, OPA1, DRP1), enhances metabolic activity and ATP production, and thereby promotes M2 polarization. It also scavenges reactive oxygen species through intrinsic SOD/catalase activity, thereby reducing oxidative damage. In osteoporotic mice, the microspheres enhanced M2 macrophage infiltration at bone defect sites and accelerated regeneration via the macrophage–miR-5106–SIK2/3–Runx2 signaling axis. With sustained VGX-1027 release over 28 days, covering the critical healing phase, this approach demonstrates substantial therapeutic potential (205).

#### 5.1.2 Regulating pro-inflammatory gene expression

Modulating macrophage phenotypic switching through inhibition of pro-inflammatory gene expression represents a viable therapeutic strategy. For instance, Cao et al. developed gelatin microspheres co-loaded with Mg^2+^ and alendronate (GMA MSs). The released Mg^2+^ suppresses M1 polarization by inhibiting the NF-κB signaling pathway, thereby improving the local inflammatory microenvironment through upregulation of IL-10 and TGF-β and downregulation of TNF-α and IFN-γ. In parallel, hydrogen (H_2_) generated during material degradation scavenges reactive oxygen species and alleviates oxidative stress, resulting in dual modulation of inflammatory and redox homeostasis. Furthermore, Mg^2+^ activates osteogenic gene expression, promoting osteogenic differentiation of MC3T3-E1 cells. *In vivo* studies demonstrated reduced osteoclast numbers at defect sites and markedly increased new bone formation (206).

In a similar approach, Arcos et al. developed mesoporous bioactive nanoparticles loaded with ipriflavone (MBNP-IP). This system was shown to suppress NF-κB expression in macrophages, reducing the secretion of pro-inflammatory cytokines such as IL-6 and TNF-α and thereby alleviating local inflammation. Its ability to promote osseointegration was further validated in OVX rabbit models (207). In parallel, Casarrubios et al. confirmed that ipriflavone-loaded nanospheres (NanoMBG-Ips) enhance VEGFR2 secretion by M2 macrophages, consequently accelerating angiogenesis in osteoporotic fracture healing (208).

#### 5.1.3 Regulate macrophage polarization

Other biomaterial-based approaches modulate macrophage polarization to improve inflammatory and immune microenvironments and enhance osteogenesis. For example, Peng et al. developed folic acid-targeted liposomes (RSV@DTPF) that employ a ROS-responsive release mechanism to scavenge reactive oxygen species while promoting M2 polarization and rebalancing cytokine profiles—specifically reducing TNF-α and increasing IL-10. These liposomes also stimulate osteogenic differentiation of bone marrow stromal cells (BMSCs) while suppressing osteoclast-related genes (e.g., *Acp*, *Mmp9*, *Ctsk*, *Traf6*), thereby significantly inhibiting osteoclastogenesis and osteoclast maturation (209).

Liu et al. designed a tantalum-based scaffold (MZIF-8-PDA@PTa) that remodels the inflammatory microenvironment by inhibiting M1 polarization and promoting M2 transition *in vitro*, while also activating the p38-MAPK pathway to upregulate osteogenic markers such as *Runx2* and *Ocn*. In OVX rat models, the scaffold markedly enhanced osseointegration (210). In a complementary approach, Zhou et al. developed an interleukin-4-loaded extracellular vesicle–gel system (IL-4/BEVs BP@GelMA) that effectively induces M2 polarization. *In vivo* results demonstrated synergistic upregulation of osteogenic factors (e.g., *Runx2, Ocn*) and angiogenic markers (e.g., *VEGF, CD31*), collectively accelerating fracture repair (211).

Furthermore, sulfated chitosan nanoparticles (Fg@SCS NPs@rhBMP-2) and gastrodin-functionalized scaffolds (PU/n-HA) have been shown to promote M2 polarization and resolve inflammation (212, 213). Additionally, PU/n-HA scaffolds effectively recruit BMSCs and enhance their activation through improved mitochondrial biogenesis and restored mitochondrial network homeostasis.

Drug delivery systems show considerable therapeutic potential; however, several limitations require attention. These include challenges in controlling release kinetics, minimizing off-target effects, and improving the physicochemical properties of drug carriers and synthetic bone matrices. Such issues currently hinder effective clinical translation. Future research should focus on developing materials with tunable release profiles—enabling both rapid and sustained drug release—and optimizing carrier systems to better navigate the complex bone microenvironment.

### 5.2 Surface modification techniques

Surface modification techniques alter implant surface properties through physical, chemical, or electrochemical methods to improve osseointegration, stimulate osteogenesis, reduce infection risks, and decrease complications (214). By functionalizing materials to modulate macrophage responses, these techniques enable improved macrophage regulation while maintaining the inherent physical properties of the base material (**Table 3**).

**Table 3:** Surface modification technology.

Material	Matrix material	Surface coating	Roughness (nm)	Cell model	Animal models		Macrophages	Osteoclast differentiation	Osteogenic differentiation	Inflammation and OS	Others	Ref.
PEEK-PDA-Sr	PEEK	Sr, PDA	47.13±2.34	BMMs, BMSCs	OVX SD rats	Defect of femur	M1↓, M2↑	TRAP↓	ALP, OPN, Runx2, Col-I, OCN↑	ROS↓, SIRT1, SOD2, CAT↑	/	(215)
SrCO3@PCL/PDA	PCL	PDA	/	BMSCs, BMDMs, RAW264.7	OVX SD rats	Skull defect	M1↓, M2↑	NFATc1, TRAP, CTSK, MMP9, DC-STAMP↓	RUNX2, COL1, SP7, ALP, BMP2, OPN, BGLAP↑	IL-1 β, TNF-α, IL-6↓; ROS↓	/	(216)
Ti-MAO/Sr/LBLWNP	Ti	Sr, LBLWNP	93.1±7.9	RAW264.7, OBs	OVX SD rats	Defect of femur	M1↓, M2↑	/	COL-I, ALP, OPG, BMP-2↑	IL-1β, TNF-α↓; TGF-β1, IL-10↑	/	(217)
Ti@PDA+Mn	Ti	Mn, PDA	33.9±1.75	BMM, BMSCs	OVX rats	Femoral bone defects	M1↓, M2↑	TRAP↓	BMP-2↑	ROS↓, SIRT1, SOD2, CAT↑	/	(221)
ABL@ZnTA	Ti	ABL@ZnTA	/	MC3T3-E1, RAW264.7	Rats infected with S. aureus	Defect of femur	M1↓, M2↑	/	RUNX2, COL1, ALP↑	iNOS, TNF-α, IL-1β↓; IL-4, IL-10, TGF-β↑; ROS↓	Anti-S. aureus infection	(223)
Ag@TiO_2_-NTs	TiO_2_-NTs	Ag	/	RAW264.7, MC3T3-E1	OVX rats	/	M1↓, M2↑	CTSK, NFATc1, c-Fos, TRAP↓	ALP, RUNX2, OCN, OPN↑	TGF-β↑, TNF-α↓	/	(222)
Ga-Ti	Ti	Ga	/	RAW264.7, hMSCs	/	/	Osteoclast differentiation↓	NFATc1, TRAP, MMP9↓	RUNX2, COL1A1,ALP↑	ROS↓	/	(224)
SPEEK@PDA-GO	SPEEK	PDA, GO	140±16.1	RAW264.7, BMMs, BMSCs	OVX SD rats	Defect of femur	M1↓, M2↑	TRAP↓	ALP, COL-1, RUNX2↑	TNF-α, IL-6↓; IL-4, IL-10↑	/	(227)
SPEEK@PDA-LAP	SPEEK	PDA, LAP	139±6.56	RAW264.7, BMMs, BMSCs	OVX SD rats	Defect of femur	Pyroptosis↓	NFATc1, c-Fos, Cath-K↓	ALP, OCN, BMP-2↑	NLRP3, IL-1β, IL-1β↓; ROS↓	/	(228)
CPC@PCL/CaCit	CPC@PCL	CaCit	/	RAW264.7, BMDM, MC3T3-E1	OVX rats	Defect of femoral condyle	M1↓, M2↑	TRAP↓	OCN, OPN↑	/	/	(225)
Ti-(BSA@GYY)	Ti	BSA@GYY NPs, PDA	55.5±5.6	BMSCs, RAW264.7	OVX SD rats	Defect of femur	Osteoclast differentiation↓	c-Fos, NFATC1, CTSK, TRAP↓	RUNX2, ALP, OCN, OPN, COL1↑	/	/	(230)
SCP-P(A)-K	CF/PEEK	KRSR, ALN	/	RAW264.7, MC3T3-E1	Rabbits with iron overload	Defect of femur	M1↓	/	ALP, RUNX-2, OPN, COL-1↑	IL-6, TNF-α, CCL-2↓; ROS↓	/	(229)
ICA-PDA@SPEEK	SPEEK	PDA, ICA	/	BMDMs, rBMSCs	OVX SD rats	Defect of femoral condyle	M1↓, M2↑	TRAP, CTSK, NFATc1↓	BMP-2↑	TNF-α, IL-6↓; IL-4, IL-10↑	VEGF↑	(226)
Ti-ALN-acBSP	Ti	acBSP, ALN	/	BMDM, MC3T3-E1, ANA-1	OVX rats	Defect of femoral condyle	“Switch-On”: M1↑; “Switch-Off”: M1↓	/	“Switch-On”: OSM↑; “Switch-Off”: ALP↑	“Switch-On”: TNF-α, GM-CFS↑; “Switch-Off”: IL-1β, TNF-α↓	“Switch-On”: VEGF↑	(232)
Ch-GNPs/c-myb-coated Ti	Ti	Ch-GNPs/c-myb	/	MC3T3-E1, BMMs	OVX rats	Tooth defect	Osteoclast differentiation↓	NFATc1, c-Fos, TRAP↓	BMP-2, BMP-7, OPG, ALP, RUNX2↑	/	/	(231)

“Switch-On”, Phase of initiation; “Switch-Off”, Phase of closure; S. aureus, staphylococcus aureus; SPEEK, Sulfonated polyetheretherketone; ICA, Icariin; ALN, Alendronate; NA, Naringin.

#### 5.2.1 Metal ion-releasing coatings

Surface modification techniques enable the functionalization of materials to release specific ions—such as Sr^2+^, Mn^2+^, Zn^2+^, and Ag^+^—thereby modulating macrophage responses.

Sr^2+^ ions effectively modulate macrophage polarization. For instance, Zhang et al. developed Sr^2+^-anchored polyetheretherketone implants (PEEK-PDA-Sr), Du et al. designed strontium carbonate scaffolds (SrCO_3_@PCL/PDA), and Wang et al. fabricated nano-wogonin-composited strontium-doped titanium (Ti-MAO/Sr/LBL WNP). *In vitro* and *in vivo* studies demonstrated that these materials sustain Sr^2+^ release, effectively suppressing pro-inflammatory M1 polarization while promoting anti-inflammatory M2 polarization in macrophages. Additionally, they enhance osteogenic differentiation and inhibit osteoclast activity (215–217). Mechanistically, Sr^2+^ activates the PI3K/Akt signaling pathway to improve mitochondrial function while inhibiting NF-κB, thereby suppressing M1 polarization (216, 218).

Mn^2+^ ions exhibit context-dependent effects on macrophage polarization. While some studies report promotion of M1 polarization (219), others describe inhibition of M1 polarization (220). These varied outcomes appear to depend on chemical speciation, delivery system characteristics, and microenvironmental signals. Wang et al. developed Mn^2+^-modified titanium implants (Ti@PDA+Mn) that suppress M1 polarization and promote M2 polarization through scavenging reactive oxygen species and upregulating antioxidant genes (including *SIRT1, SOD2*, and *CAT*). This approach also effectively enhances osteogenic differentiation while inhibiting osteoclastogenesis (221).

Furthermore, silver nanotube coatings (Ag@TiO_2_-NTs) developed by Wang et al. enable sustained Ag^+^ release, which enhances macrophage autophagy and suppresses the NF-κB pathway (222). Separately, zinc-peptide metal-phenolic nanocoatings (ABL@ZnTA) designed by Xu et al. release Zn^2+^ under infectious conditions, inhibiting iNOS and TNF-α expression while promoting IL-10 and TGF-β secretion in macrophages, thereby achieving both antibacterial and osteogenic outcomes (223).

Leveraging the similar ionic behavior between Ga^3+^ and Fe^3+^, Piñera-A et al. modified titanium surfaces with gallium-doped perovskite layers. The released Ga^3+^ binds to transferrin and enters cells via transferrin receptor 1. This uptake competitively inhibits cellular iron absorption, disrupts iron metabolism, and accelerates the Fenton reaction, promoting ROS accumulation and ultimately leading to ferroptosis. Consequently, this process inhibits macrophage differentiation into osteoclasts (224). These functionalized coatings typically exhibit biphasic ion release profiles, characterized by an initial burst followed by a sustained release phase. Such release kinetics help suppress bacterial infection in early stages while providing continuous stimulation for bone regeneration in later phases.

#### 5.2.2 Regulate metabolic reprogramming

Certain biomaterials modulate immune responses by regulating macrophage metabolism. For example, Wu et al. developed citrate-functionalized scaffolds (CPC@PCL/CaCit). Citrate released from these scaffolds binds to specific sites on key glycolytic enzymes, thereby inhibiting their activity and redirecting metabolic flux toward the TCA cycle. This metabolic shift enhances oxidative phosphorylation, supporting an M2-like polarization state (225).

#### 5.2.3 Anti-inflammatory coatings

Certain materials function through anti-inflammatory mechanisms. For instance, Chai et al. developed an icariin-modified sulfonated polyetheretherketone system (ICA-PDA@SPEEK), where the loaded icariin exhibits anti-inflammatory properties by promoting M2 macrophage polarization, reducing pro-inflammatory cytokine secretion, and increasing anti-inflammatory factor production (226). Likewise, sulfonated polyetheretherketone modified with graphene oxide (SPEEK@PDA-GO) or laponite (SPEEK@PDA-LAP) inhibits macrophage pyroptosis and osteoclast activation through suppression of the STAT3-mediated NLRP3/caspase-1/IL-1β signaling axis (227, 228).

#### 5.2.4 Coatings that inhibit osteoclast differentiation

Another category of functional coatings functions by suppressing osteoclast differentiation from macrophages. Zhou et al. designed an intelligent supramolecular coating (SCP-P(A)-K) that releases the anti-resorptive drug alendronate (ALN) to inhibit osteoclastogenesis (229). Similarly, Xia et al. developed a titanium-based system (Ti-(BSA@GYY)) that suppresses osteoclast differentiation through modulation of the OPG/RANKL pathway (230). Additionally, Takanche et al. reported that Ch-GNPs/c-myb nanoparticles inhibit RANKL signaling and the JNK pathway while blocking NF-κB nuclear translocation, thereby effectively preventing osteoclast differentiation of macrophages (231).

#### 5.2.5 Temporally regulated macrophage modulation coatings

An emerging strategy involves the temporal regulation of macrophage function through smart coating design. Wang et al. developed a Ti-ALN-acBSP coating capable of sequentially regulating macrophage responses. During the early phase, the coating releases acidic bone sialoprotein (acBSP) to polarize macrophages toward the M1 phenotype. This establishes an appropriate inflammatory microenvironment while promoting the secretion of osteogenic factors such as oncostatin M and pro-angiogenic factors including VEGF, thereby initiating repair processes. In later stages, alkaline phosphatase secreted by osteoblasts triggers coating degradation, which induces apoptosis of pro-inflammatory macrophages, resolving local inflammation (232). This sequential immunomodulatory strategy better replicates the physiological shift in macrophage phenotypes during normal bone healing, providing a promising framework for designing biomaterials with temporally controlled macrophage-regulating properties.

### 5.3 Hydrogels

As localized delivery carriers, smart hydrogels enable dynamic release of active components in response to microenvironmental changes, while providing tunable mechanical properties and high biocompatibility (**Table 4**).

**Table 4:** Hydrogel material.

Material	Composition	Cell model	Animal models		Macrophages	Osteoclast differentiation	Osteogenic differentiation	Inflammation and OS	Others	Ref.
ROS-scavenging hydrogel	EGCG, APBA	/	OVX rabbits	Defect of femur	M1↓, M2↑	/	RUNX2, ALP, COL-1, BSP, OPN, OCN↑	IL-1β, IL-6↓; IL-4, IL-10, TGF-β1↑; ROS↓	VEGF↑	(237)
MnO2@Pol/HA	Poloxamer 407, HA, MnO2 NPs	/	OVX rabbits	Radius defect	M1↓, M2↑	/	RUNX2, ALP, COL-1, OCN↑	TNF-α, IL-1β, IL-6↓; TGF-β, PDGF↑; ROS↓	/	(236)
PESATO/MCys-Ca	SA-SATO, PEGDA, Mcys, Ca	RAW264.7, BMSC, HUVEC	OVX SD rats	Defect of femur	M1↓	RANKL, TRAP↓	OPG, RUNX 2, BMP-2, ALP, OCN↑	ROS↓	New blood vessels↑	(234)
GelMA-QK/Sr-LDH@PDA	Sr-LDH@PDA, GelMA, QK peptide	BMMs, BMSCs, bEnd.3	OVX SD rats	Defect of femoral condyle	Proliferation↑, Osteoclast differentiation↓	CTSK, MMP9, c-Fos, NFATc1↓	ALP, RUNX2, COL-1, OPN↑	/	CD31, vWF, PDGFF-BB↑	(241)
FAPI-MMS-Gel	MMS, FAPi, m-PGA/GelMA	BMMs, BMSCs	OVX SD rats	Defect of femoral condyle	M1↓, M2↑	c-Fos, NFATc1, ACP5, MMP9,CTSK↓	ALP, SPP1, RUNX2, COL-1, BGLAP↑	IL-1β, TNF-α↓; IL-10, TGF-β↑; SIRT1, SOD2, NRF2↑; ROS↓	/	(233)
DEX@CHAp/Res@CHAp/Col I/PLEL	PLEL, CHAp, DEX, RES, Col-I	RAW264.7, BMSCs, HUVESs	OVX SD rats	Defect of femur	M1↓, M2↑	/	RUNX2, ALP, OCN, BGLAP, SPP1↑	TNF-α↓, ROS↓	New blood vessels↑	(235)
PEG/nHAp/CS	tetra-PEG, nHAp, CS	BMMs, RAW 264.7	OVX SD rats	Skull defect	M1↓, M2↑	/	RUNX2, OCN↑	IL-1β↓, IL-10↑	/	(240)
SDF-1α/CS/GP/HEC	CS, GP, HEC	BMSCs	OVX rats	Alveolar bone defect	M1↓, M2↑	/	RUNX2, OCN, ALP, BMP1, COL-1↑	IL-1β, TNF-α, INOS↓; IL-10, Arg1↑	/	(239)
Gel/Alg-Kae aerogel	GelMA, Alg, KAE	RAW264.7, BMSCs, HUVECs, BMMs	OVX rats	Defect of femur	M1↓, M2↑	c-Fos, MMP9↓	RUNX2, ALP, OPN, OCN↑	ROS↓	New blood vessels↑	(238)

EGCG, Epigallocatechin-3-gallate; APBA, 3-Acrylamido phenylboronic acid; HA, Hyaluronic acid; SA-SATO, Sodium Alginate-S-aroylthiooxime; PEGDA, Poly(ethylene glycol) diacrylate; Mcys, ROS-responsive microspheres containing cysteine; MMS, MnO_2_-coated CaP microspheres; FAPi, Fibroblast Activation Protein Inhibitor; PLEL, poly(D,L-lactide)-poly(ethylene glycol)-poly(D,L-lactide; CHAp, Carbonated hydroxyapatite microspheres; DEX, Dexamethasone; tetra-PEG, extra-armed poly(ethylene glycol); nHAp, Nano-hydroxyapatite; CS, Short-chain chitosan; GP, β-Glycerophosphate; HEC, Hydroxyethyl Cellulose; KAE, Kaempferol.

#### 5.3.1 Antioxidant hydrogels

Numerous hydrogel systems demonstrate notable antioxidant properties. For instance, Chen et al. developed a methacrylated gelatin hydrogel incorporating a fibroblast activation protein inhibitor (FAPI) and MnO_2_ nanoparticles (FAPI-MMS-Gel). Both *in vitro* and *in vivo* studies confirmed that FAPI-MMS-Gel reduces reactive oxygen species (ROS) levels in bone marrow stromal cells and bone marrow macrophages (BMMs) by upregulating antioxidant genes such as SIRT1 and SOD2, while also suppressing the NF-κB pathway to promote M2 macrophage polarization (233). JJiang et al. designed an H_2_S-sustained-release hydrogel (PESATO/MCys-Ca) and demonstrated that its ROS-responsive H_2_S release significantly decreases intracellular ROS in macrophages, inhibits M1 polarization and osteoclast-related genes (e.g., *Acp5*, *Mmp9*), and upregulates osteogenic markers (e.g., *Runx2*, *Ocn*) (234). Li et al. developed a thermosensitive resveratrol/dexamethasone gel that effectively scavenges DPPH radicals and promotes macrophage transition from M1 to M2 phenotype (235). Other systems, including MnO_2_@Pol/HA (Ye et al.), EGCG/APBA composite hydrogel (Ding et al.), and kaempferol aerogel (Jin et al.), also efficiently eliminate ROS, promote the shift from M1 to M2 polarization, and reduce pro-inflammatory cytokine levels (e.g., TNF-α, IL-1β, IL-6) (236–238). Collectively, these antioxidant hydrogels alleviate inflammatory and oxidative stress, reduce M1 polarization while enhancing M2 polarization, thereby establishing a more favorable microenvironment for osseointegration in osteoporotic fractures and bone defects.

#### 5.3.2 ROS-independent hydrogels

Some hydrogel materials function independently of ROS modulation. For example, Liu et al. developed an SDF-1α-functionalized chitosan hydrogel that promotes BMSCs migration and osteogenic differentiation *in vitro*, while suppressing M1 polarization and enhancing M2 macrophage polarization *in vivo* (239). Sun et al. designed a PEG/nHAp/CS hydrogel that inhibits M1 polarization and promotes M2 polarization by antagonizing the TLR4/NF-κB pathway, thereby shifting the local microenvironment toward an anti-inflammatory state. This hydrogel also enhances osteogenesis via the cAMP/PKA/CREB pathway. In OVX Sprague-Dawley rats with calvarial defects, implantation with PEG/nHAp/CS hydrogel increased the proportion of CD206^+^ M2 macrophages, decreased CD86^+^ M1 macrophages, and markedly reduced osteoclast numbers (240). He et al. developed an Sr-LDH/GelMA-QK hydrogel that promotes macrophage proliferation, suppresses osteoclast differentiation, enhances endothelial cell migration, and upregulates vasculogenesis-related genes (e.g., *CD31, vWF*). Animal studies demonstrated that this material inhibits osteoclast maturation, increases PDGF-BB release, and promotes H-type vessel formation to support osseointegration in osteoporotic bone (241). Additionally, Li et al. designed a Gel-Ale-Mg@PDA nanocomposite scaffold that induces M2 macrophage polarization through controlled Mg^2+^ release and promotes osteogenic differentiation of BMSCs (242).

While hydrogel-based strategies demonstrate therapeutic potential, further optimization is needed for hydrogel compositions and the controlled-release kinetics of bioactive molecules. Future studies should also clarify the complex interactions among hydrogels, macrophages, and other cell types during bone regeneration, particularly to address challenging pathological conditions (18, 243).

### 5.4 Biological ceramics

Although bioceramics were previously the main focus of research, current studies on bioceramic-assisted osseointegration have progressively shifted toward composite materials incorporating bioceramics (**Table 5**).

**Table 5:** Bioceramics, alloys and other materials.

Biological ceramics									
Material	Cell models	Animal models		Macrophages	Osteoclast differentiation	Osteogenic differentiation	Inflammation and OS	Others	Ref.
PKNN	RAW264.7, rBMSCs-OVX	/	/	M1↓, M2↑	/	RUNX2, ALP↑	TNF-α, IL-1, IL-6↓; Arg-1, IL-4, IL-10, Tgfb1↑	/	(244)
Strontium silicate	L929, hMSCs, RAW 264.7	/	/	M1↓, M2↑	/	/	NO↓, Urea↑	/	(247)
PTHrP-1-TBC	BMSCs, BMMs, HUVECs, RAW264.7	SD rats	Skull defect	M1↓	TRAP, CTSK, RANK, NFATc1↓	ALP, RUNX2, OCN↑	IL-1β, iNOS, CCR7↓	BFGF, VEGF ↑	(246)
La-LDH	rBMSCs-OVX, BMMs	OVX rats	Skull defect	Osteoclast differentiation↓	NFATc1, c-Fos, CTSK, CTR, TRAP, V-ATPase d2, DC-STAMP↓	ALP, Runx-2, OCN, COL-I, OPG↑	/	/	(245)
Sodium alendronate loaded poly(L-lactide-co-glycolide) microparticles immobilized on ceramic scaffolds	MG-63, PBMCs	Minipigs	Tooth extraction socket	Osteoclast differentiation↓	/	/	/	/	(249)
MBG-PCL-zol	Saos-2, RAW264.7	OVX ovine	Long bone defect	Osteoclast differentiation↓	/	/	/	/	(251)
RGO/ZS/CS	mBMSCs, RAW264.7, HUVECs	/	/	Osteoclast differentiation↓	TRAP, NFATc1, MMP9, CAR2↓	ALP, COL-1, CON, RUNX2↑		VEGF, BFGF, eNOS↑	(248)
NanoMBG-75S	RAW 264.7, J774A.1	/	/	M2↑	/	/	IL-6↓, TNF-α↑, ROS↓	/	(250)
Alloy									
Zn-2Cu-0.5Zr	RAW264.7, BMSCs, MC3T3-E1	SOP rats	Tibial fracture	M1↓, M2↑	/	ALP, COL-1, RUNX2, OSX, OPN, OCN↑	TNF-α, IL-6, IL-1β↓; IL-10↑; ROS↓	/	(202)
Ti6Al4V-Cu	BMMs-OVX	/	/	M1↓, M2↑	/	/	IL-1β, IL-6, TNF-α↓; IL-10↑	/	(252)
Ti6Al4V-6wt.%Cu	BMMs-OVX	/	/	M1↓, M2↑	/	CTSK, NFATc1, TRAP↓	IL-1β, IL-6, TNF-α↓; IL-10↑	/	(253)
Bone cement									
Mg-BG-BC	BMSCs, RAW264.7	/	/	M1↓, M2↑	/	RUNX2, ALP, OCN, OPN, OSX ↑	CXCL-11, NOS2, IL-1β↓; Arg-1, CCL-17↑	/	(254)
CMC/OPC	BMSCs, RAW264.7	BALB/C mice	/	M1↓, M2↑	/	RUNX2, OPN, COL-1↑	/	/	(255)
Others									
G-O-A@Mg	RAW264.7, BMSCs	OVX SD rats	Femoral condyle defect	M1↓, M2↑	TRAP↓	RUNX2, OCN↑	/	/	(256)
Picein	BMSCs, RAW264.7, HUVECs	OVX rats	/	M2↑	/	ALP, BMP4, COL-1↑	IL-1β, IL-6, TNF-α↓; ROS↓	New blood vessels↑	(257)

PKNN, Polarized Potassium Sodium Niobate Ceramic; rBMSCs-OVX, These cells were bone marrow mesenchymal stem cells isolated from OVX rats; Strontium silicate, SrSiO3 and Sr_2_SiO4 are included; La-LDH, Lanthanum-substituted MgAl layered double hydroxide nanohybrid scaffolds-A novel ceramic material with biological activity; MBG-PCL-zol, Mesoporous Bioactive Glass/ϵ-Polycaprolactone/Zoledronic Acid scaffolds; RGO/ZS/CS, Reduced graphene oxide/zinc silicate/calcium silicate; SOP, Senile Osteoporosis; CMC/OPC, Carboxymethyl cellulose-reinforced calcium phosphate/calcium sulfate cement; G-O-A@Mg, Gelatin-Oxidized dextran-Alendronate@Magnesium-A bone cement.

For example, Wang et al. developed a piezoelectric PKNN ceramic that maintains stable piezoelectric performance under physiological conditions, effectively reduces M1 macrophage polarization markers, promotes M2 phenotypic transition, and synergistically enhances osteogenic differentiation of BMSCs via the Runx2 pathway (244). Chu et al. designed a La-LDH nanohybrid scaffold that inhibits osteoclast differentiation of BMMs through suppression of NF-κB signaling, while upregulating the OPG/RANKL ratio to stimulate osteogenesis (245). The PTHrP-1-TBC scaffold enables sustained release of PTHrP-1, which inhibits M1 polarization and activates the pro-angiogenic factor VEGF, thus establishing a synergistic pro-angiogenic and osteoinductive microenvironment (246). Furthermore, strontium silicate particles (SrSiO_3_/Sr_2_SiO_4_) reduce M1 polarization and enhance M2 polarization, thereby promoting BMSCs migration (247). Xiong et al. developed a conductive RGO/ZS/CS scaffold that promotes BMSCs osteogenesis via silicon/zinc ion release and inhibits macrophage-derived osteoclastogenesis; its extracts significantly upregulate angiogenic genes (*VEGF, bFGF*) in HUVECs, thereby enhancing neovascularization (248). Rumian et al. immobilized AlN-loaded PLGA microparticles on a ceramic scaffold to form a composite material that inhibits osteoclast differentiation of macrophages (249). Feito et al. developed NanoMBG-75S, which reduces oxidative stress and promotes M2 polarization of macrophages (250). Meanwhile, Gómez-Cerezo et al. designed an MBG-PCL-zol scaffold that effectively inhibits osteoclastogenesis; however, *in vivo* studies indicate that high local concentrations of released zoledronic acid may trigger inflammatory responses, requiring further optimization (251).

Most bioceramic implants demonstrate favorable biocompatibility and bioactivity. Combined with modern additive manufacturing techniques, they allow precise design of pore structure and morphology, enhancing their functional versatility.

### 5.5 Alloy materials

Alloy materials represent a well-established class of implants used to enhance osseointegration (**Table 5**). For instance, Ji et al. developed a zinc-based alloy (Zn-2Cu-0.5Zr) in which released Zn^2+^ significantly reduces ROS levels in macrophages and suppresses pro-inflammatory cytokine secretion, while also activating the Wnt/β-catenin signaling pathway. Both *in vitro* and *in vivo* studies confirmed that this material promotes M2 macrophage polarization and accelerates bone regeneration (202). In another approach, copper-modified titanium alloy (Ti6Al4V-Cu) upregulates COMMD1 to inhibit NF-κB phosphorylation, thereby reshaping the macrophage polarization balance within inflammatory microenvironments. Concurrently, it suppresses osteoclast differentiation and promotes osteoblast-derived extracellular matrix (OBECM) formation (252, 253).

Alloy implants are widely employed in fracture and bone defect repair owing to their excellent mechanical properties and the capacity of released metal ions to exert antibacterial and osteogenic effects. Current developments in implant alloys emphasize biodegradable designs to avoid secondary surgical procedures. However, several challenges remain for biodegradable alloy materials. For instance, a standardized system for evaluating the cytotoxicity of degradable metals *in vitro* has not yet been established. The properties and biological effects of alloys vary with the types, proportions, and microstructure of constituent metals, making extensive experimental validation necessary to identify optimal compositions. In addition, the mechanical properties of such materials gradually decline during degradation, which may restrict their use in load-bearing sites and in the context of delayed fracture healing.

### 5.6 Other materials

Bone cement and certain specialized compounds have been shown to facilitate the repair of osteoporotic fractures or bone defects by regulating macrophage polarization (**Table 5**). For example, Dai et al. developed a magnesium-doped bone cement (Mg-BG-BC) that promotes the transition of macrophages from the M1 to the M2 phenotype through Mg^2+^ release and modulation of the TLR/MyD88 signaling pathway, while also enhancing the osteogenic differentiation of BMSCs (254). Separately, Li et al. introduced a novel bone cement formulation (CMC/OPC) that effectively induces M2 polarization of macrophages (255). In another approach, Zhao et al. constructed a G-O-A@Mg porous adhesive loaded with Mg^2+^ and alendronate, which modulates macrophage polarization while inhibiting osteoclast differentiation (256).

Furthermore, Huang et al. demonstrated that the natural compound picein (C_14_H_18_O7) exhibits notable anti-inflammatory and antioxidant properties. It promotes M2 macrophage polarization, suppresses ferroptosis in BMSCs, and enhances osteogenic differentiation through activation of the Nrf2/HO-1/GPX4 signaling pathway (257).

Several natural compounds have demonstrated potential in ameliorating osteoporosis and enhancing fracture healing, with certain compounds exhibiting modulatory effects on macrophage function. For instance, maltol—a natural compound derived from red ginseng—alleviates postmenopausal osteoporosis by promoting RNF213-mediated ubiquitination of CDK14 in macrophages, thereby suppressing M1 polarization and reducing TNFSF12-induced osteoblast apoptosis (258). Similarly, naringenin promotes M2 polarization of macrophages and reduces the secretion of pro-inflammatory factors, promoting bone formation while inhibiting bone resorption, thereby ameliorating pathological bone loss (259). Although these studies did not jointly analyze osteoporosis, fracture healing, and macrophage regulation, they suggest that such natural compounds may facilitate the healing of osteoporotic fractures and bone defects through macrophage-mediated mechanisms.

In addition, combining such natural compounds with other biomaterials represents a viable approach to broaden their therapeutic applicability and enhance treatment efficacy. For example, Zhou et al. developed a dual-targeted nanoplatform that delivers baicalein to fracture sites, where it reduces inflammation, promotes osteogenic differentiation of BMSCs, and accelerates fracture healing by inducing macrophage M2 polarization (260). Separately, Pan et al. constructed a PU/n-HA scaffold for sustained gastrodin release, thereby modulating macrophage responses and facilitating bone repair (213). These collective findings support the potential of natural compounds—either alone or integrated with biomaterials—as a macrophage-targeting strategy worthy of further investigation for managing osteoporotic fractures.

## 6 Challenges and future directions

Although macrophage-targeted therapies hold promise for treating OPF, their clinical translation faces several challenges. While current evidence indicates that macrophage metabolic reprogramming and polarization influence OP progression, the precise impact of key OP-inducing factors—such as aging and estrogen deficiency—on these cellular processes remains incompletely characterized in existing experimental models. Moreover, the spatiotemporal heterogeneity, polarization transitions, and metabolic adaptations of macrophage subpopulations during OPF healing have yet to be fully elucidated, requiring more systematic and high-resolution analysis in physiologically relevant settings.

Emerging research on the gut-bone and neuro-osteal axes has advanced our understanding of osteoporosis pathogenesis. However, their crosstalk with macrophage-mediated bone metabolism—particularly in the context of OPF—is not yet fully understood. Furthermore, the expression dynamics of macrophage subtypes and inflammatory mediators during fracture healing under osteoporotic conditions, such as in postmenopausal or aging models, have not yet been fully characterized. Deeper mechanistic studies are essential to clarify these regulatory networks and establish a solid foundation for future therapeutic development.

During normal fracture healing, macrophages require a coordinated transition from the pro-inflammatory M1 phenotype to the reparative M2 phenotype. While current therapeutic strategies primarily focus on promoting M2 polarization and suppressing M1 polarization, achieving optimal repair outcomes likely requires precise temporal regulation of macrophage phenotypic switching. Experimental evidence indicates that although existing approaches enhance osseointegration, greater therapeutic efficacy could be attained through dynamic control of polarization timing. For example, the Ti-ALN-acBSP coating enables temporal regulation of M1 macrophage responses through cellular crosstalk involving macrophages, OBs, and OCs, providing a promising paradigm for future temporally controlled strategies (232). However, most currently available biomaterials lack the capacity for precise spatiotemporal control over macrophage polarization, which may result in suboptimal bone regeneration and limited clinical efficacy in osteoporotic bone defects (205).

Concerning experimental models, most current studies on osteoporotic fractures and bone defects utilize animal models—particularly ovariectomized rats—to simulate human OPF conditions. However, these models do not fully recapitulate the human condition due to interspecies differences in both local microenvironment and mechanical properties, which may limit their translational relevance. Moreover, while ovariectomy-based models are widely used to simulate postmenopausal osteoporosis, research focusing on macrophage regulation in aged models remains limited, despite the higher clinical incidence of OPF in elderly populations. The pathogenic mechanisms underlying estrogen deficiency-induced osteoporosis differ substantially from those of age-related bone loss, which may influence the therapeutic efficacy of biomaterial-based interventions (203). Additionally, surgically created fractures and bone defects may not accurately mimic the pathophysiological processes of spontaneous osteoporotic fractures. These collective limitations reduce the clinical predictive value of current animal models and affect the generalizability of resulting findings (261).

Future studies should integrate spatial metabolomics with single-cell epigenetic sequencing to delineate the metabolism–epigenetics interaction network of macrophage subpopulations in OPF. Such multi-omics integration would improve understanding of macrophage dynamics and functional regulation during OPF healing, thereby informing targeted therapeutic strategies. For instance, Xue et al. demonstrated that combining single-cell RNA sequencing with spatial metabolomics can effectively elucidate how drug treatments alleviate tissue damage by reshaping macrophage metabolism and promoting M2 polarization. Their study provides a methodological paradigm for linking transcriptional programs with spatial metabolic microenvironments to unravel disease mechanisms (262). Adopting such an integrated approach in OPF research will help uncover the metabolic–epigenetic regulatory network of macrophages and offer reliable insights for precise immunomodulatory therapy.

Furthermore, more clinically relevant animal models of osteoporotic fracture should be established. These could include aging-based OPF models or patient-derived xenograft systems involving transplantation of bone marrow from elderly osteoporosis patients into immunodeficient mice, better replicating the human “inflammaging” microenvironment. Alternatively, advanced organoid models incorporating osteoporotic bone matrix, vascular networks, and patient-derived macrophages could be developed to enable longitudinal monitoring of microenvironmental dynamics during OPF healing.

## 7 Conclusions

In summary, this review systematically outlines the immunometabolic regulatory network of macrophages in OPF and its therapeutic relevance. We have highlighted how OP-related pathological factors—such as estrogen deficiency and aging—impair macrophage metabolic reprogramming and polarization dynamics through mechanisms including glycolysis/OXPHOS imbalance, succinate accumulation, and NAD^+^ deficiency, ultimately contributing to delayed fracture healing. A range of emerging macrophage-targeted strategies are thoroughly discussed, spanning metabolic regulatory nanocrystals (e.g., ZIF-H_2_S-SDSSD), temporally responsive implant coatings (e.g., Ti-ALN-acBSP), immunomodulatory hydrogels, and functionally enhanced biomaterials. These interventions facilitate bone regeneration by remodeling the osteoimmune microenvironment and rebalancing osteogenic and osteoclastic activities.

However, several research gaps remain: the spatiotemporal distribution of macrophage subsets during OPF healing is not yet fully mapped; existing aging-related OPF models exhibit translational limitations; insights into cross-system interactions such as the gut-bone axis and their link to macrophage metabolism are still insufficient; and most therapeutic systems lack precise temporal control over the M1-to-M2 transition.

Future research should prioritize the integration of spatial metabolomics with single-cell multi-omics profiling to establish more accurate inflammaging models, investigate novel neuro–immune and gut–bone regulatory targets, and develop smart biomaterials capable of dynamically guiding macrophage metabolism and polarization. Such advances will be crucial for translating immunometabolic precision therapies into clinical OPF management.

## Glossary

acBSP: Acidic bone sialoprotein
ACOD1: Aconitate decarboxylase 1
ALN: Alendronate
BCAA: Branched-chain amino acid
BCKDH: Branched-chain α-keto acid dehydrogenase
BMMs: Bone marrow macrophages
BMSCs: Bone marrow stromal cells
CXCL: C-X-C motif chemokine ligand
DBT: Dihydrolipoamide branched-chain transacylase E2
DPF: Days post-fracture
ETC: Electron transport chain
FAO: Fatty acid oxidation
FAPI: Fibroblast activation protein inhibitor
Fas: Fatty acids
GO: Graphene oxide
LAP: Laponite
mtROS: Mitochondrial ROS
NADPH: Nicotinamide adenine dinucleotide phosphate
NAM: Nicotinamide
NaMN: Nicotinic acid mononucleotide
NAMPT: Nicotinamide phosphoribosyltransferase
NGF: Nerve growth factor
NMN: NAM mononucleotide
OBs: Osteoblasts
OCs: Osteoclasts
OP: Osteoporosis
OPF: Osteoporotic fracture
OVX: Ovariectomized
PGE2: Prostaglandin E2
PPP: Pentose phosphate pathway
RANKL: Receptor Activator of Nuclear Factor Kappa-B Ligand
ROS: Reactive oxygen species
SCFA: Short-chain fatty acids
SDH: Succinate dehydrogenase
SOD: Superoxide dismutase
SOP: Senile osteoporosis
TCA: Tricarboxylic acid
TrkA: Tropomyosin receptor kinase A
TMAO: Trimethylamine oxide
VEGF: Vascular endothelial growth factor
